# Digital protractor as an intraoperative guide to cup anteversion in total hip arthroplasty

**DOI:** 10.1186/s12893-023-02297-9

**Published:** 2024-01-18

**Authors:** Ning Yi, Hang Yuan, Nanwei Xu, Ruiping Liu, Yuji Wang, Chao Zhuang

**Affiliations:** 1 Graduate School of Dalian Medical University, Dalian, China; 2 Bengbu Medical College, Bengbu, China; 3 Department of Orthopedics, The Affiliated Changzhou Second People’s Hospital of Nanjing Medical University, Changzhou, China

**Keywords:** Digital protractor, Anteversion, Total hip arthroplasty, Direct anterior approach

## Abstract

**Introduction:**

To explore if digital protractor could guide the anteversion of acetabular cup during primary THA and make it consistent with preoperative.

**Methods:**

We retrospectively reviewed 172 cases of primary THA with direct anterior approach (DAA) over 2 years. The anteversion of acetabular cup were measured from computed tomography (CT) scan preoperative and de-identified plain radiographs postoperative by two blinded investigators who were not involved in the surgery. The effect of the digital protractor on the anteversion was determined using regression analysis.

**Results:**

The mean anteversion for the THAs in digital protractor group was 15.5°and 21.4°in control group (*P* < 0.01). The mean anteversion bias for the THAs in digital protractor group was 1.59° and 6.63° in control group (*P* < 0.01).Regression analysis identified a 10.7% difference in anteversion due to the use of digital protractor (*P* < 0.01), and THAs performed without digital protractor were six times more likely to result in anteversion of > 25°. The correlation coefficient for the interobserver reliability of the measurement of the two investigators was 0.94.

**Conclusion:**

The digital protractor is a practical tool in the DAA for THA to determine the anteversion of the acetabular prosthesis.

## Introduction

In total hip arthroplasty (THA), incorrect placement of the acetabulum cup will lead to postoperative prosthesis dislocation, wear rate increase, early loosening, and other complications [1]. Proper placement of the acetabular prosthesis can reduce these risks and help to promote successful clinical outcomes [2].

The currently widely cited “safe zones” concept for the placement of the acetabular cup, which is defined as 5°to 25°anteversion and 30°to 50°abduction angle, was proposed by Lewinnek et al. in 1978. When the cup was placed in this interval, the prosthesis dislocation rate was only 1.5%. When the cup was placed outside this interval, the prosthesis dislocation rate was 6.1% [3].

There are several techniques that can help surgeons perform optimal acetabular cup positioning, including mechanical guidance, but these methods can only ensure a moderate degree of accuracy [4, 5]. The inaccuracy of traditional placement may be due to several reasons, such as the surgeon’s estimation of errors, changes in the anatomical structure of the patient, and the change of the pelvic position during the operation [6, 7]. Using the conventional posterior lateral approach, the hip dislocation and the use of the retractor can cause pelvic obliquity and change the pelvic position, which may lead to a misjudgment of the placement of the acetabular prosthesis [8].

The direct anterior approach (DAA) for THA was first described by Hueter and was popularized in France in early 1947 [9, 10]. This surgical approach can operate in the internerve and intervascular plane without stripping the muscles. In the past 10years, DAA has attracted wide attention. Compared with the traditional posterior lateral approach, DAA has the advantage of not invading the integrity of the external rotator muscles of the hip, thereby reducing the risk of dislocation after THA [11, 12]. Pain after DAA is less compared with the posterior lateral approach. Compared with the posterior lateral approach, the hip joint function and gait ability recovered quickly after DAA [13, 14]. Additional, the anteversion of the acetabular prosthesis can be measured directly during surgery using a magnetic instrument referring to the horizontal line.

The purpose of this research was to determine whether the use of digital protractor affected the anteversion of the acetabular cup and if its use reduced the prosthesis anteversion bias.

## Materials and methods

We retrospectively reviewed 172 consecutive femoral neck fracture patients who undergo primary THAs using cement or non-cement prosthesis but all metal balls at our hospital from January 2017to December 2018. All patients signed informed consent regarding publishing their data. Half of the procedures were performed with digital protractor (86 patients) and half were without (86 patients).

**Fig. 1:**
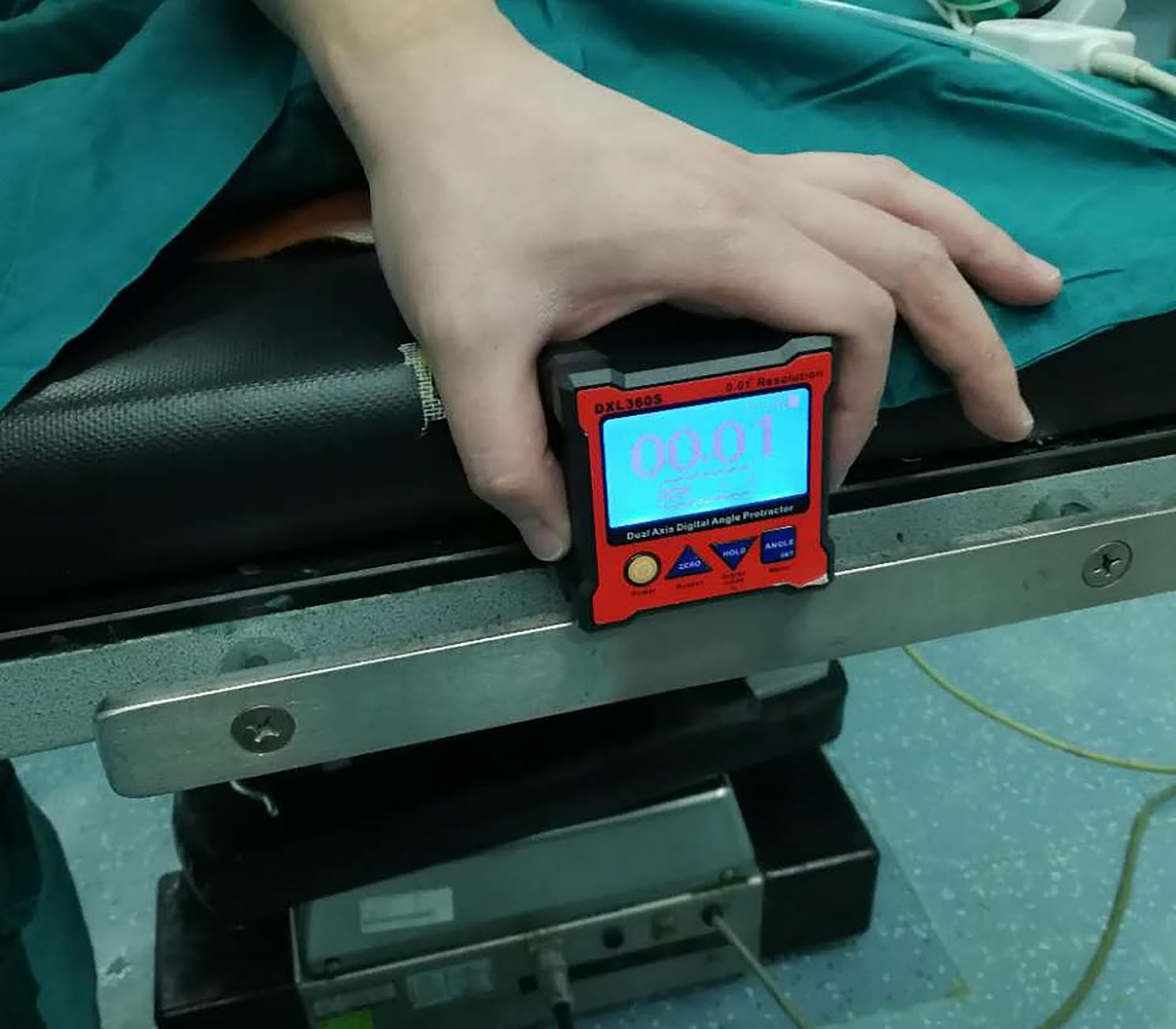
The operating table is positioned horizontally to the floor

**Fig. 2:**
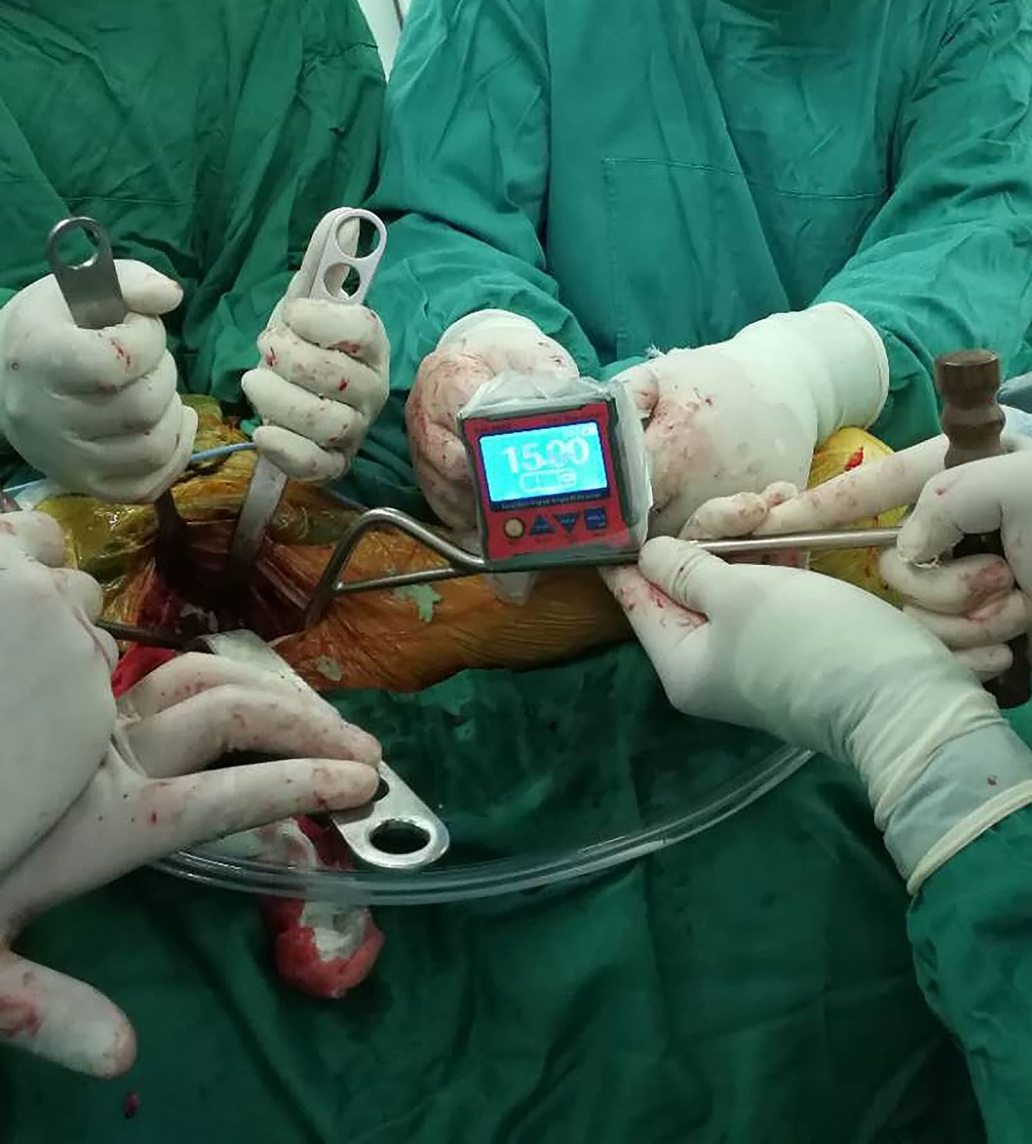
The acetabular cup is placed, and the digital protractor is placed on the metal bar to adjust and maintain an anteversion of 15°

Our operative technique for DAA is slightly modified. The patients were in the supine position on the standard orthopedic operating tablewhich is horizontal (Fig. 1).According to the DAA technique, the acetabulum was exposed by removing the acetabular labrum and the osteophyte. The ligamentum capitis femoris was removed, and the oval fossa was exposed. The acetabulum was reamed, the digital protractor was placed on the cup introducer to adjust and maintain the anteversion of the acetabulum which is according to the CT scan preoperative until the component was seated (Fig. 2).The anteversion of the acetabulum was assessed visually in the control group, by judging the angle between the handle of the cup introducer and the floor.

A standardized cross-table lateral view of the postoperative hip was taken with the contralateral lower extremity flexed as much as possible to eliminate lumbar lordosis and place the pelvis in a standard position. All films were coded and given an ID by the coordinator of the study, who was the only person with access to the key. The anteversion of the acetabular component was measured according to the technique of Woo [15]by two blinded investigators who had not been involved in the operations.

The mean anteversion and anteversion bias for the digital protractor and control groups were initially compared using a two-tailed Student’s t-test. The effect of use of the digital protractor on the anteversion was determined with multiple regression analysis and the effect of its use on placement in the ‘safe zone’, between 5° and 25°, was evaluated with multiple logistic regression analysis. Patient-specific demographics, including age, gender, BMI, the side of the operation and prosthetic types were accounted for in all regression analyses.The interobserver reliability of the radiographic measurements was determined using an intraclass correlation coefficient(ICC). Statistical significance was set at *P* < 0.05, and all analyses were performed by an author involved in neither the operations nor the measurement of the angles. All statistical analyses were conducted using SPSS 22.0 software (SPSS Inc., Chicago, IL, USA).

## Results

All patients had successful surgery. The drainage tubes were removed 2 days after surgery, and the patients were allowed to walk with walkers. The radiographic examination was performed, and the anteversion of the acetabular prosthesis was measured 3 to 7 days after surgery. The patients were discharged from the hospital 7 to 10 days after the operation. No case of prosthesis dislocation and other complication occurred during the hospital stay and the follow-up period (11.7 ± 5.3 months)after discharge in either group.

**Fig. 3:**
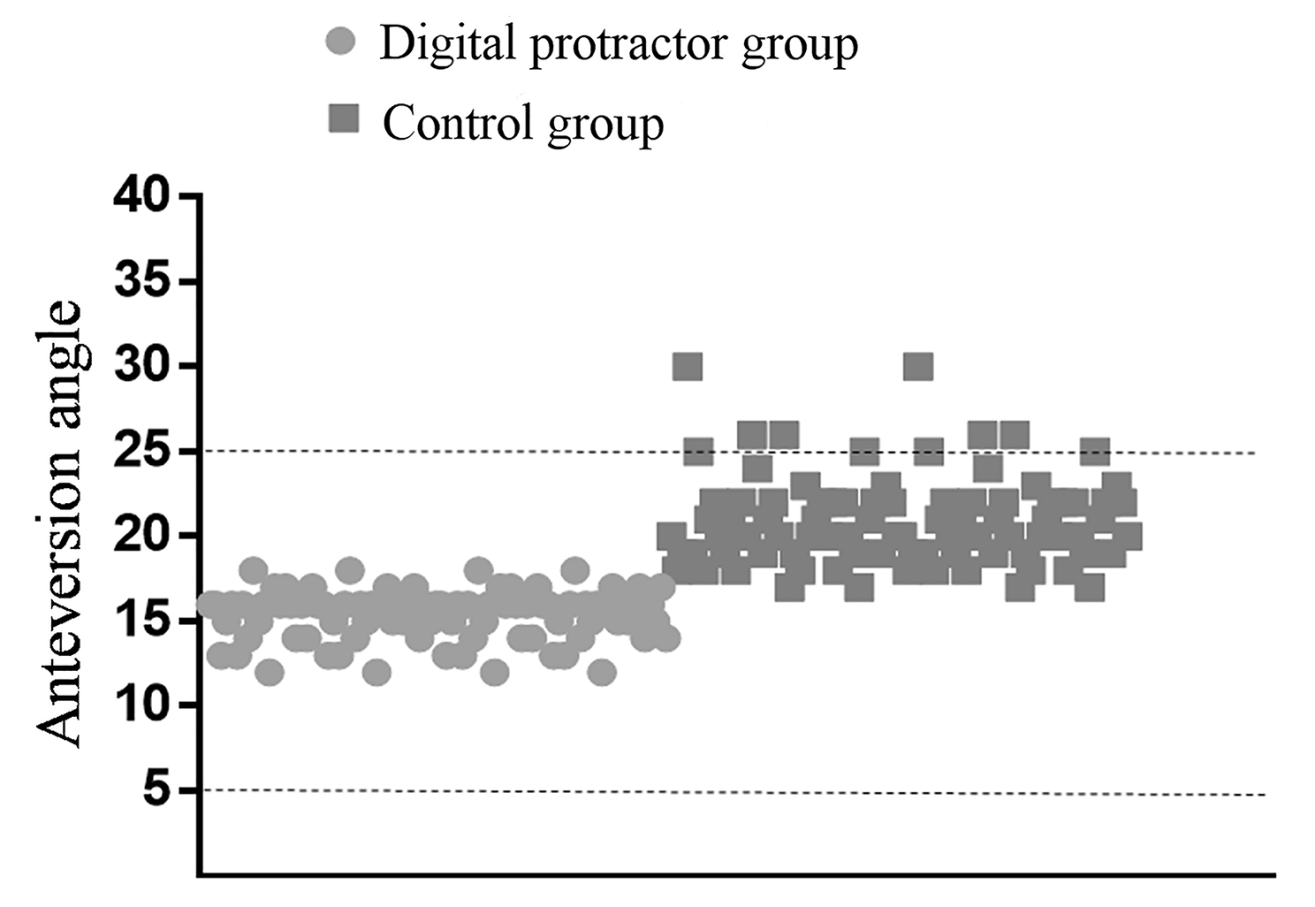
Scatterplot showing angles with and without the digital protractor. Light grey, digital protractor group; Dark grey, control group. The dashed lines specify the “safe zone” of 5° to 25°

The demographics of the patients were similar in both groups (Table 1). Mean radiographic anteversion for the 86 THAs in the digital protractor group was 15.5° (95% confidence interval (CI) 15.1° to 16.0°; range 12° to 20°) compared with 21.4° (95% CI 20.6° to 22.2°; range17° to 30°) for the 86 in the control group (*P* < 0.01). The average anteversion bias for the THAs in digital protractor group was 1.59°(95% CI 1.03° to 1.96°; range 0° to 4°) and 6.63° (95% CI 5.46° to 7.85°; range 3° to 15°) in control group (*P* < 0.01).A total of 6 (6.9%) in the control group had an angle of > 25° but none in the digital protractor group (Fig. 3). Linear regression analysis showed a 10.7% difference in anteversion when the digital protractor was used (*P* < 0.01).Regression analysis showed that placement of the prosthesis outside the “safe zones” was almost six to one, with measurement of > 25° being more likely in the control group (OR 5.4, *P* = 0.027).The ICC for interobserver reliability was 0.94 (95% CI 0.92 to 0.96).

**Table 1:** Patient demographics and mean anteversion for each cohort

	Digital protractor group(n = 86)	Control group (n = 86)	*P*-value
Age	71.8 ± 9.4	72.3 ± 8.5	0.374^a^
Gender(male/female)	30/56	25/61	0.532^b^
Body mass index(BMI)	27.34 ± 3.25	26.52 ± 2.91	0.279^a^
Operation side(left/right)	48/38	45/41	0.532^b^
Prosthetic types			0.683^b^
Cement	23	22	
Cemetless	63	64	
Radiographic anteversion(°) preoperation	13.8 ± 1.1	14.1 ± 1.4	0.244^a^
Radiographic anteversion(°) postoperation	15.5 ± 1.6	21.4 ± 2.9	0.003^a*^
Anteversion bias(°)	1.59 ± 0.98	6.63 ± 2.28	0.0012^a*^

^a^Student’s *t*-test

^b^Chi-squared test

^*^Statistically significant

## Discussion

Usually, the positioning of the acetabulum cup is achieved by the manufacturer’s alignment guide in THA. However, this method often leads to incorrect orientation of the prosthesis, because the pelvic position changes during the routine posterior lateral approach [16–18]. Some authors reported that compared with the inclination angle, the anteversion of the acetabular cup using the conventional positioning method was more inaccurate [7, 19]. Unlike the inclination angle, most scholars tend to be approximately45°, there is no consensus on how much anteversion angle the acetabulum cup requires [20, 21].At present, the computer navigation system seems to be the most reliable location tool if initial registration is accurate. However, the use of such a system in general hospitals is limited because of its high cost [22–24].Other scholars have also reported the use of the transverse acetabular ligament as an anatomical sign to determine the anteversion of the prosthesis [25, 26]. However, we have found that some elderly patients have an unrecognizable degeneration of the transverse acetabular ligament, and whether the cup edge is parallel to the acetabular transverse ligament or placed a certain angle is still an academic problem. The correct position of the acetabular prosthesis in THA directly affects the range of hip movement, polyethylene wear, and pelvis osteolysis, which are very important in reducing the risk of dislocation [27, 28]. To achieve a reliable alignment, there is a need for technical aids and tools based on reproducibility.

The DAA is a modification of the Smith-Pedersen method, because only the distal part of the incision is used. This technology became the regular technique of Judet and Judet in 1947 [29, 30]. In THA, if the posterior lateral approach is taken, the patient is in the lateral position, and the pulling force traction of the acetabulum can make the pelvic tilt angle change greatly, which will lead to an error of the position of the acetabular cup. DAA is the most superficial approach to the exposure of the hip joint. The acetabulum is relatively easy to expose and does not need to be widely used as a traction device; thus, the pelvic tilt angle will not change during the surgery. In the supine position, the pelvis can be fixed horizontally on the operating table, and the tilting angle of the pelvis is also smaller when the acetabulum cup is fixed.

In our study, we realize that the anteversion is not a static parameter, but a specific dynamic value for each individual. Therefore, we use the preoperative CT scan to determine the patient’s anteversion. During the surgery, the acetabular prosthesis is placed in this angle. During our 172 primary THAs with the DAA, we found that accuracy of placement was improved when a digital protractor was used, with fewer prosthesis being put outside the ‘safe zone’ of between 5°and 25°, which present a simple and reproducible way of obtaining the desired anteversion.

There are limitations in this study. Its retrospective design limits its strength. The reason why CT measurement was not used postoperatively was due to metal artifacts, but X-ray was indeed not as accurate as CT. Further, we did not include an analysis of the abduction of the acetabular component, which is an important determinant of anteversion. Also, this study only included patients with femoral neck fracture, but did not study patients with osteopathy. Because the anteversion of patients with osteopathy often deviates from normal physiological value, we often need to correct the anteversion during operation to make it return to ‘safe zone’, which is inconsistent with our research plan, so they are excluded. Ceramic prostheses are not included in this study, because according to our experience, ceramic prostheses need to be placed in a larger anteversion (25°–30°), which is not in line with the research program, so they are excluded.

## Conclusion

Measuring the anteversion of an acetabular prosthesis using the digital protractor through DAA in THA is a reliable technique that is worthy of clinical promotion.

## Data Availability

Data available on request and should be contacted with corresponding author.
